# Most accurate mutations in SARS-CoV-2 genomes identified in Uzbek patients show novel amino acid changes

**DOI:** 10.3389/fmed.2024.1401655

**Published:** 2024-05-31

**Authors:** Mirzakamol S. Ayubov, Mukhammadjon K. Mirzakhmedov, Abdurakhmon N. Yusupov, Akmal M. Asrorov, Bakhtiyor V. Nosirov, Dilshod E. Usmanov, Shukhrat E. Shermatov, Khurshida A. Ubaydullaeva, Abdusattor Abdukarimov, Zabardast T. Buriev, Ibrokhim Y. Abdurakhmonov

**Affiliations:** ^1^Center of Genomics and Bioinformatics, Academy of Sciences of Uzbekistan, Tashkent, Republic of Uzbekistan; ^2^Department of Chemistry for Natural Substances, National University of Uzbekistan, Tashkent, Uzbekistan; ^3^Luxembourg Institute of Health, Strassen, Luxembourg

**Keywords:** SARS-CoV-2, virus, transmission, AA mutations, delta strain

## Abstract

**Purpose:**

The rapid changes in the coronavirus genomes created new strains after the first variation was found in Wuhan in 2019. SARS-CoV-2 genotypes should periodically undergo whole genome sequencing to control it because it has been extremely helpful in combating the virus. Many diagnoses, treatments, and vaccinations have been developed against it based on genome sequencing. With its practical implications, this study aimed to determine changes in the delta variant of SARS-CoV-2 widespread in Uzbekistan during the pandemic by genome sequencing, thereby providing crucial insights for developing effective control strategies that can be directly applied in the field.

**Design:**

We meticulously generated 17 high-quality whole-genome sequence data from 48 SARS-CoV-2 genotypes of COVID-19 patients who tested positive by PCR in Tashkent, Uzbekistan. Our rigorous approach, which includes stringent quality control measures and multiple rounds of verification, ensures the accuracy and reliability of our findings.

**Methods:**

Our study employed a unique combination of genome sequencing and bioinformatics web tools to analyze amino acid (AA) changes in the virus genomes. This approach allowed us to understand the genetic changes in the delta variant of SARS-CoV-2 widespread in Uzbekistan during the pandemic.

**Results:**

Our study revealed significant nucleotide polymorphisms, including non-synonymous (missense) and synonymous mutations in the coding regions of the sequenced sample genomes. These findings, categorized by phylogenetic analysis into the G clade (or GK sub-clade), contribute to our understanding of the delta variant of SARS-CoV-2 widespread in Uzbekistan during the pandemic. A total of 134 mutations were identified, consisting of 65 shared and 69 unique mutations. These nucleotide changes, including one frameshift mutation, one conservative and disruptive insertion-deletion, four upstream region mutations, four downstream region mutations, 39 synonymous mutations, and 84 missense mutations, are crucial in the ongoing battle against the virus.

**Conclusion:**

The comprehensive whole-genome sequencing data presented in this study aids in tracing the origins and sources of circulating SARS-CoV-2 variants and analyzing emerging variations within Uzbekistan and globally. The genome sequencing of SARS-CoV-2 from samples collected in Uzbekistan in late 2021, during the peak of the pandemic’s second wave nationwide, is detailed here. Following acquiring these sequences, research efforts have focused on developing DNA and plant-based edible vaccines utilizing prevalent SARS-CoV-2 strains in Uzbekistan, which are currently undergoing clinical trials.

## Introduction

Considerable research has been conducted on the SARS-Cov-2 genome since 2020. To date, 15,778,185 SARS-CoV-2 genome sequences have been shared via online data platforms such as GISAID (1), which helps track the new variants and mutations. The causative agent of COVID-19, SARS-CoV-2, is constantly evolving as it spreads from human to human (2).

On 11 March 2020, the World Health Organization (WHO) formally declared a pandemic (3). SARS-CoV-2 has scattered globally and is currently a significant problem. More people have suffered in Europe and Latin America than in other countries, especially during the early phases of the coronavirus outbreak.

Scientists are currently using the established nomenclature systems for naming and tracking genetic lineages of SARS-CoV-2 through GISAID, Nextstrain, and Pango in their research. The WHO called for the creation of the Technical Advisory Group on Virus Evolution, which suggested labeling the virus strains with Greek alphabet letters, such as Alpha, Beta, Gamma, Delta, etc., to facilitate more accessible and more helpful discussion among audiences outside of the scientific community (3).

SARS-CoV-2 is classified within the β-coronavirus genus lineage B. It is characterized by possessing large enveloped, positive-sense, single-stranded RNAs with a length of 30 kilobases. The viral genome encodes four structural proteins and various accessory and non-structural proteins. Notable among these are the viral pp1a-pp1ab replicase, the 3C-like protease (3CLpro), the papain-like protease (PLpro), and the RNA-dependent RNA polymerase (RdRp) (4, 5).

Finding relevant information on viral lineages, variants of interest, and variants of concern requires SARS-CoV2 whole-genome sequencing (6). Global databases of the SARS-CoV-2 genome have been mentioned in our previous paper (7).

The virus genomes sequenced were used to create the global phylogenetic tree of SARS-Cov-2. The sequences are categorized into multiple clades. Our previous study examined the earliest clades and their characteristics (7). Many viral variants were later discovered and uploaded to global databases. The Delta variant was first reported in India at the end of 2020, but it has since spread worldwide to 135 countries and continues growing. Delta shared some mutations with other variants and possessed unique mutations on spike proteins, which may be responsible for its rapid spread and increased virulence (8). Significantly more transmissible delta variants have been connected to important S-protein mutations like D614G, L452R, P681R, and T478K (9). The spike protein promotes virus-cell membrane fusion and attachment to host cell-surface receptors. Additionally, it serves as the main target for neutralizing antibodies made following infection (10).

For the first time, an infectious epidemic was found in Uzbekistan in March 2020. The government decided to conduct public testing for COVID-19. This incident signaled the beginning of the pandemic in Uzbekistan, necessitating accurate identification and monitoring of coronavirus genotypes that were rapidly spreading throughout the population (7, 11). The SARS-CoV-2 genome should be sequenced at the population level to identify virus strains and investigate their local and worldwide dissemination. In addition, the comprehensive sequencing of the entire viral genome associated with infection is a valuable tool in elucidating outbreak dynamics. This includes investigating variations in pandemic size over time, spatial dissemination patterns, and transmission routes, as emphasized in the WHO COVID Report of 2021.

The global distribution of the virus was caused by worldwide travel. We started sequencing the whole genome of COVID-19 samples to primarily identify virus genotypes spread in our territory and analyze genomic diversity, types of mutations, and the emergence of new variations of SARS-CoV-2 (7, 11). In this study, we described the following whole-genome sequence data from individuals in the Republic of Uzbekistan who were infected with COVID-19. We evaluated 134 mutations, including non-synonymous and synonymous, and effectively reconstructed 17 high-quality sample genome sequences for COVID-19 genotypes. In the global phylogenetic tree, comparative analysis utilizes SARS-CoV-2 genomes known as the Uzbekistan sample genomes in the GK subclade. The S region of the sequenced genomic data of one of the coronavirus genotypes was used to develop the edible vaccine against coronavirus threats in Uzbekistan (12).

## Materials and methods

### Gathering samples

Using the technique from our earlier research (7), samples were taken from 100 symptomatic patients with a high temperature and an intermittent cough. The samples were then immediately put in a viral transport medium. Patients suspected of having COVID-19 infection were referred to the diagnostics laboratories of the private BiogenMed COVID-19 testing laboratory in Tashkent, Republic of Uzbekistan. After SARS-CoV-2 testing in the laboratory, specimens of biological origin were randomly collected from PCR-positive individuals.

The scientific inquiry was approved by the Ethics Committee of the Ministry of Health of the Republic of Uzbekistan (#6/20-1582). All experiments were conducted in conformity with the applicable standards and legislation. Samples were renumbered and de-identified so that no one, not even researchers, knew who the patients were. Only anonymized data, including age and biological sex, were preserved for reporting purposes. All patients in the sample collection provided verbal consent for voluntary participation. We stated to all participants that the collected samples would be used for a sequencing project exclusively, without disclosing their identities or disturbing them in the future. In this study, verbal consent was preferred over written consent since patients were hesitant to sign any written document due to their concern about COVID-19 infection at the time and a lack of knowledge of the genome sequencing investigation. No minors were involved in the sample collection. There was no need to do so because the sequencing experiment was non-invasive, participants were not subjected to further downstream clinical procedures, and the identity of samples was anonymized in this investigation.

RNA extraction, Real-Time PCR, and sample evaluation were carried out after our previous work (7). Among all evaluated patients, 48 PCR-positive samples (28 females and 20 men) were randomly chosen for further research.

### SARS-CoV-2 sequencing

SARS-CoV-2 sequencing and data analysis were carried out as in our previous study (8). We used coverage analysis (v5.12.0.0) and variant caller (v5.12.0.4) while estimating the probability of the data belongings. Variant callers were filtered to retain only dependable variants and eliminate variants with reading depths of less than 1000 and ion torrent quality scores of less than 400 (Table 2 and Supplementary Table 1). We utilized the Maximum Likelihood Tree in the Molecular Evolutionary Genetics Analysis (MEGA)^1^ program to cluster samples for the filtered variants. The consensus for each SARS-CoV-2 genome sequence was then submitted to the NCBI under accession numbers GI:2085183815 to GI:2085183892 (or MZ892621.1 to MZ892627.1; NCBI database; Table 1) and to GISAID^2^ under accession numbers EPI_ISL_3668627 to EPI_ISL_3673673 (available for registered users).

**TABLE 1:** An overview of the COVID-19 samples used in the present study.

ID	Collection date	Sex	Age	Coverage	GISAID accession #	NCBI accession #	Clade
**10**	23/07/2021	Female	54	5943	EPI_ISL_3668627	MZ892621.1	GK
**33**	23/07/2021	Female	36	2854	EPI_ISL_3668631	*	GK
**34**	23/07/2021	Female	24	17146	EPI_ISL_3673667	*	GK
**35**	23/07/2021	Female	22	854,5	EPI_ISL_3673670	MZ892622.1	GK
**36**	23/07/2021	Female	6	5729	EPI_ISL_3673672	*	GK
**37**	23/07/2021	Male	58	3305	EPI_ISL_3668628	MZ892623.1	GK
**38**	23/07/2021	Female	11	3167	EPI_ISL_3668632	MZ892624.1	GK
**39**	23/07/2021	Male	33	1813	EPI_ISL_3673666	*	GK
**40**	23/07/2021	Female	59	2856	EPI_ISL_3668633	*	GK
**41**	23/07/2021	Male	31	3134	EPI_ISL_3673668	*	GK
**42**	23/07/2021	Female	38	1874	EPI_ISL_3673671	*	GK
**43**	23/07/2021	Female	13	1807	EPI_ISL_3668629	MZ892625.1	GK
**44**	23/07/2021	Male	35	3442	EPI_ISL_3673673	*	GK
**45**	23/07/2021	Male	50	1339	EPI_ISL_3668634	*	GK
**46**	23/07/2021	Female	29	3448	EPI_ISL_3668630	MZ892626.1	GK
**47**	23/07/2021	Female	47	2819	EPI_ISL_3673669	*	GK
**48**	23/07/2021	Female	28	3309	EPI_ISL_3668635	MZ892627.1	GK

*GISAID only has accession numbers for these samples, but not in NCBI. Only registered users have access to the sequencing data provided to the GISAID database.

## Results and discussion

### Sample selection for sequencing

More than a hundred patients suffering from high fever and sporadic cough symptoms were randomly selected in this work from a Tashkent-based private COVID-19 testing laboratory. Forty-eight PCR-positive samples were chosen for sequencing. Only 17 high-quality sequences were selected to be submitted to worldwide databases (twelve women and five men with an average age of 34, Table 1).

The results in this work show twice the higher infectivity level of women after random analysis. Twelve of seventeen patients were women among those subjected to SARS-CoV-2 infection during random selection. Thus, we expect greater sensitivity of women among Uzbekistani patients than men to SARS-CoV-2 infection. We did not observe the tendency of older people to be more sensitive to coronavirus infection. Among the studied patients, only four out of seventeen people were >50 years old after random selection. The Delta variant is notably more transmissible, estimated to have a 50–80% increase in transmissibility compared to the Alpha variant (13). In addition, the Delta variant is believed to have higher transmissibility compared to earlier strains of SARS-CoV-2, particularly among children and adolescents. According to a decision analytic model analyzing 106,866 confirmed COVID-19 infections, susceptibility to the Delta variant was significantly higher among the 10 to 15-year-old age group, showing a 1.92-fold increase compared to the pre-Delta variant (14). This corresponds with our results: seven patients were <30 years old after random selection.

There are numerous gaps in the consensus sequence as a result of 31 SARS-CoV-2 samples being disqualified from additional analysis due to inadequate sequencing coverage. The average number of mapped reads for the remaining 17 samples (12 women and five men) was 779 739 (Table 1). The target reads were 99.61%, and the mean read depth was 3814. The average coverage consistency in the chosen samples was 87.8% (Table 2).

**TABLE 2:** Selected high-quality sequenced samples along with high coverage.

#	Sample name	Mapped reads	Target reads (%)	Mean depth	Uniformity (%)
1	CGB-10	1121237	99.64	5943	87.93
2	CGB33	518399	99.84	2854	87.54
3	CGB-34	3133616	99.76	17146	90.83
4	CGB-35	201758	99.46	854.5	92.67
5	CGB-36	1151910	99.76	5729	80.04
6	CGB-37	620105	99.83	3305	80.50
7	CGB-38	789833	99.41	3167	89.13
8	CGB-39	442640	99.48	1813	90.51
9	CGB-40	506837	99.81	2856	78.00
10	CGB-41	714637	99.47	3134	92.57
11	CGB-42	436501	99.52	1874	91.55
12	CGB-43	431153	99.48	1807	91.85
13	CGB-44	643427	99.88	3442	73.85
14	CGB-45	279054	99.67	1339	89.57
15	CGB-46	806289	99.45	3448	90.32
16	CGB-47	665638	99.43	2819	91.42
17	CGB-48	792525	99.44	3309	93.81

### Identifying unique and reliable mutations in all high-quality sequenced samples

**FIGURE 1:**
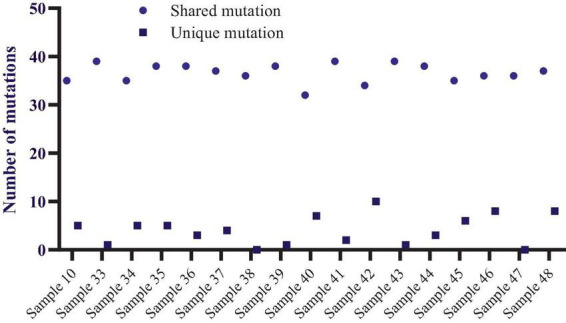
The number of reliable mutations in sequenced SARS-CoV-2 genomes.

Using the maximum likelihood method, the MEGA X program generated a phylogenetic tree based on the 17 viral sequences of SARS-CoV-2 taken from samples of COVID-19 patients in Tashkent city. The Wuhan reference strain NC 045512.2 was the root of this maximum likelihood tree, which was constructed using 134 mutations found in the 17 sequences (Supplementary Table 1 and Figure 1). Bootstrap values are illustrated.

Variant Caller produced all evaluated mutations in these selected samples. The number of mutations ranged from 36 (samples 38,47, and 36) to 45 (sample 48), altogether unique and shared mutations (Figure 1). Compared to the NC_045512.2 reference genome, most viral genomes represented between 39 and 44 mutations. These included thirty-nine mutations in samples 39 and 40, forty mutations in samples 10, 33, 34, and 43, forty-one mutations in samples 37, 41, 44, and 45, and forty-three mutations in only samples 35 and forty-four mutations in samples 42 and 46. The mutations mentioned above were calculated together with shared and unique mutations. Samples 38 and 46 do not carry unique mutations (Figure 1).

The total number of shared mutations and unique mutations is calculated for each sample.

The most commonly occurring nucleotide substitution found was cytosine to thymine (51/134 mutations), then guanine to thymine (28/134 mutations), thymine to cytosine and guanine to adenine (12/134 mutations), and then adenine to guanine (10/134 mutations). The Supplementary Table 1 shows the breakdown of these mutations. Every mutation had homozygosity. In the gene encoding the S protein we identified one shared disruptive inframe deletion (c.467_472delAGTTCA), two unique (c.*4300A>G, c.*4352G>T), two shared (c.*4308G>T, c.*4358G>T) downstream gene variant mutations, four unique (c.646C>T, c.2533G>T, c.3761G>T and c.3790G>T) and nine shared missense variant mutations, four unique (c.936C>T, c.1995C>T, c.2259G>A and c.2388T>C) and one shared (c.3183C>T) synonymous variant mutations. In the M (matrix) region one unique (c.100C>T and c.463C>T) and one shared (c.245T>C) missense mutation was observed; in N (nucleocapsid) region one shared (c.-3delA) upstream gene variant mutation, three unique (c.200C>T, c.518C>T and c.1085C>T) and six shared (c.188A>G, c.608G>T, c.643G>T, c.1129G>T, c.1152G>C and c.1154G>A) missense mutations and four unique (c.105G>A, c.123G>A, c.987G>A and c.1080C>T) synonymous mutations were identified. No mutations were found in the E (envelope) region (Supplementary Tables 1, 2). The nucleotide substitution C>T was established to enhance the viral adaptation in hosts (15). The reason was linked with the occurrence of amino acids of a hydrophobic nature that could contribute to better cell penetration. This change was reported as evidence of host-dependence and approach to combat coronavirus (16, 17).

In addition, our analysis revealed one frameshift mutation, 41 missense mutations, 27 synonymous mutations, and two upstream gene mutations within the ORF1ab region. Within the ORF3a region, one synonymous mutation and ten missense mutations were identified. In the ORF6 region, one missense mutation was observed, while the ORF7a region displayed six missense mutations and one synonymous mutation. Additionally, the ORF8a region exhibited one missense mutation, one upstream gene mutation, and one conservative insertion-deletion mutation. Finally, one synonymous mutation (c.57C>T) was found in the ORF10 region. We found 134 mutations in total, 65 shared and 69 unique, representing one frameshift mutation, one conservative and disruptive inframe deletion, four upstream region mutations, four downstream region mutations, 39 synonymous mutations, and 84 missense mutations (Supplementary Table 1).

### The construction of the phylogenetic tree was derived from viral sequences

We wanted to examine the significant alterations in all sequences to understand what distinguished our cases from others worldwide. The software MEGA X was employed to construct a phylogenetic tree utilizing the maximum likelihood method, incorporating the analysis of 17 viral sequences (Figure 1). The variant caller identified one hundred thirty-four mutations from the abovementioned sequences (v.5.12.0.4) (Supplementary Table 1).

Most of the shared mutations with more sequences, for example, eighteen mutations (C3037T, G15451A, C16466T, C21618G, GAGTTCA22028G, T22917G, C22995A, A23403G, C23604G, G24410A, C25469T, AGATTTC28247A, T26767C, TA28270T, A28461G, G28881T, G29402T, and G29742T) with 17 sequences and four mutations (G210T, C241T, C14408T, T27638C, C27752T) with 16 samples, seven mutations (C6402T, C8986T, G9053T, A11201G, A11332G, C19220T and G28916T) with 12 sequences and four mutations (C14925T, A21137G, A24110C and A25439C) with six sequences, accordingly (Supplementary Table 1).

These variants have been grouped into the USA, Indian, and England variants. All are considered Delta strains (B.1.617.2) in the phylogenetic tree provided by genomedetective.com. Interestingly, Beta variant samples are located in the Neighbor Cluster in the tree, whereas Alpha, Gamma, and Omicron variant samples were found in the further branches (Figure 3).

This phylogenetic tree was generated in www.genomedetective.com using our sequenced samples.

*B.1.1.7 I20 belongs to the Alpha variant. B.1.351 20H belongs to the Beta variant. P.1 20J belongs to the Gamma variant. B.1.617.2 21A, 21I, 21J belong to the Delta variant. BA.1 21K, BA.2 21L, BA. 1/BA.2, BA.4 22A, and BA.5 22B belong to Omicron variant of SARS-CoV-2.

Our findings show that the whole-genome sequences obtained from the 17 symptomatic COVID-19 patients represent significant nucleotide diversity (Table 1 and Supplementary Tables 1, 2). Based on the similarity of mutations observed in the Delta strain, the 17 sequenced samples have been clustered into a predominant clade of SARS-CoV-2 within the GISAID public database, designated as clade G (or GK subclade). The G clade has been known for S-protein mutations such as D614G, L452R, P681R, and T478K [11]. These mutations were also found in all 17 samples using the viralvar.org website. However, some other mutations in this region were found in each sample. For example, 34 S-protein mutations were identified in sample 39, while 23 mutations were found in sample 47 (Supplementary Table 3).

In accordance with the findings of Cherian et al. (18), the recently identified lineages B.1.617.1 and B.1.617.2 were prominently represented within the phylogenetic tree. These lineages were characterized by signature mutations, including L452R, T478K, E484Q, D614G, and P681R, within the spike protein, notably within the receptor-binding domain (RBD) (18). Those mutations, except E484Q, were also shared with all 17 samples in our result. In addition, mutation T19R was shared with all samples, whereas G142D (in 15 samples), D950N (in 12 samples), I850L (in 6 samples), T95I (in 5 samples), A222V and F797C (in 2 samples) were found (Supplementary Table 3).

The structural analysis of the receptor-binding domain (RBD) mutations, specifically L452R, T478K, and E484Q, indicated a potential enhancement in ACE2 binding affinity. Conversely, the presence of P681R within the furin cleavage site suggested a possible acceleration in the rate of S1-S2 cleavage, thereby facilitating heightened transmissibility. Moreover, the RBD mutations L452R and E484Q were found to diminish the binding affinity to specific monoclonal antibodies (mAbs), potentially impacting their neutralization efficacy (18). Interestingly, the E484Q mutation did not appear in our sequenced samples.

**FIGURE 2:**
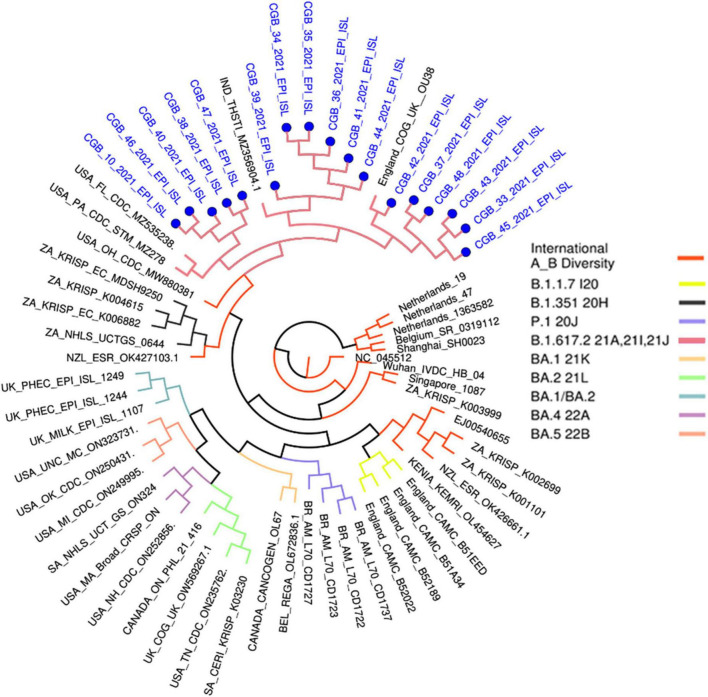
Coronavirus typing tool analysis result.

Most of the unique amino acid changes were found in the S-spike region in Sample 39 (26 mutations; F106L, G107V, T108L, T109L, L110stop, D111I, S112R, K113R, T114P, Q115S, S116P, L117Y, I119L, V120L, N121I, N122T, A123L, T124L, N125M, V126L, V127L, I128L, V130S, C131V, E132N, and Q134N) and in Sample 47 (16 mutations; A260V, G261W, A262C, A264S, Y265L, Y266L, V267C, Y269L, L270S, A263C, L277I, Q271S, P272T, R273stop, T274D, and L276S). In contrast, five AA changes were found in Sample 33 (Supplementary Table 3). The virus genome must be sequenced to identify SARS-CoV-2 strains and investigate local and worldwide dissemination. In addition, the comprehensive sequencing of the entire viral genome associated with infection is a valuable tool in elucidating outbreak dynamics. This includes investigating variations in pandemic size over time, spatial dissemination patterns, and transmission routes, as emphasized in the WHO COVID Report of 2021. Indeed, this investigation revealed that many diseases originated in the United States, India, and England (GK subclade) (Figure 2). The global distribution of the virus was caused by worldwide travel.

The disparities observed in virus structure among the samples illustrate the evolution of viral morphology over time in response to varying environmental conditions. The genomic sequence data derived from these 17 samples, subsequently deposited in global databases such as NCBI and GISAID, hold significant promise for public health and research entities in elucidating patterns of disease dissemination within the region. Moreover, these findings are anticipated to inform the development of diagnostic assays, therapeutic interventions, and vaccine candidates. Several institutions, including our center, have initiated efforts toward developing a national vaccine based on the SARS-CoV-2 genome sequences identified in symptomatic patients from Uzbekistan. The sequence information presented herein enriches the COVID sequence database (GISAID) with updated sequence data and mutational profiles specific to our geographical region. This contribution holds significance for forthcoming molecular epidemiological investigations and evolutionary phylogenetic studies conducted by health and scientific organizations.

The structural map of SARS-CoV-2 proteins was developed based on the one by Jamison et al. (19). The proteins’ 3D structures were developed by modeling their sequences in Swissmodel.^3^ The ones with no crystals obtained yet were not modeled in the database; thus, we did not include their models.

**FIGURE 3:**
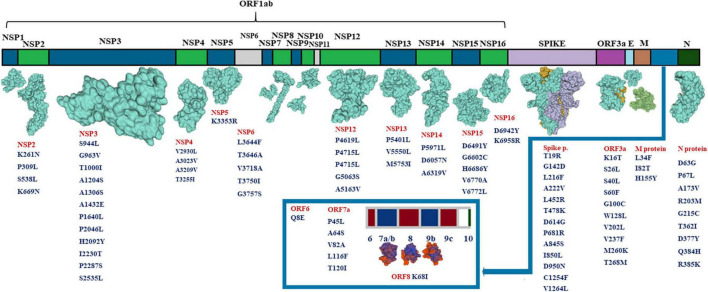
Mutations determined in patients studied in this work.

The mutations observed in this work belonged to both structural and non-structural proteins (Figure 3). Among non-structural proteins, we did not determine changes in NSP1, NSP7, NSP8, NSP9, NSP10, and NSP11. The number of mutations observed in this work coincides with proteins’ molecular parameters; the highest number of mutations were determined in NSP3 and spike protein. NSP3, the largest coronavirus protein, is one of the essential proteins involved in replication/transcription processes (20). NSP3 is one of the well-established proteases in SARS; NSP3 is responsible for papain-like functions (21) and NSP5 is known as 3CL (chymotrypsin-like main) protease (22).

Compared to protein length, the highest numbers of mutations in the genome were observed in N protein (9 mutations) and ORF3a (10 mutations). Even though the highest numbers (12 and 13) of mutations were found in NSP3 and spike proteins, respectively, their frequencies were intermediate relative to protein length. Compared to previous variants, the delta variant has higher transmissibility, and the efficacy of vaccines was lower against it (9). We needed to carry out whole genome reads to understand the genome changes better and fight those new variants. Our results demonstrated twice higher missense mutations than synonymous ones.

### Spike protein

The high pathogenic effects of coronavirus during the pandemic were linked to many features. The affinity receptor-binding domain (RBD) of spike protein to human angiotensin-converting enzyme 2 (ACE2) was one of the most cited points contributing to the deadly effects of the virus (23). Mutations in the RBD in various virus strains determined its impact on ACE2, leading to reduced or enhanced binding affinity (24). In this work, we observed six mutations (plus one deletion) in all studied 17 patients (Table 1). Only four changes were determined in one patient. Two mutations were determined in 2 and 6 patients (Table 3).

**TABLE 3:** Types of mutations detected in spike protein among 17 studied patients in Uzbekistan.

Missense mutations detected in all 17 patients					
T19R	L452R	T478K	D614G	P681R	D950N
**Missense mutations detected in one patient only**					
L216F	A845S	C1254F	V1264L		
**Missense mutations in some patients but not all**					
A222V (2 patients)		I850L (6 patients)		G142D (10 patients)	

**FIGURE 4:**
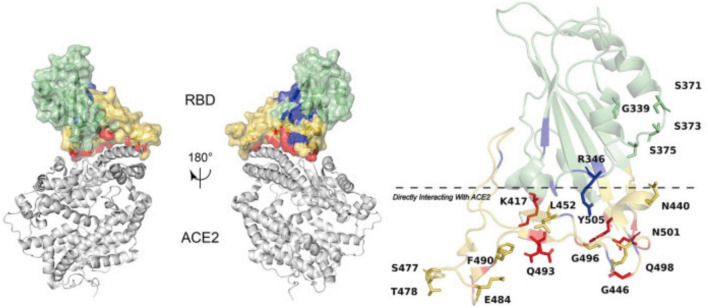
Amino acid mutations established in spike protein RBD and the RBD affinity with ACE2. The figure has been re-used from (28) with the permission of Frontiers.

The changes D614G, L452R, P681R, and T478K were earlier detected in other works (25, 26). Among these changes, the ones causing the changes in electrostatic interactions on the surface can be considered the most significant. Surface charge is regarded as one of the points that could contribute to spike protein binding efficacy based on electrostatic interaction that might facilitate conformational changes (27, 28). Among the patients studied in this work, we observed several changes that did not affect the affinity of the RBD of the spike protein to ACE2. Most of the changes either belonged to sites other than RBD or were not included in RBD-ACE2 interactions. Three of six mutations detected in all patients in this work were changes to either Arg or Lys. The significant change belonged to pLeu452Arg, where the Leu at 452 position changed to Arg. Aggarwal et al. (29) established enhanced binding affinity resulting from the pLeu452Arg mutation (29). This mutation was earlier found in Indian patients (18, 28). The location of Leu at the 452 position is described in Figure 4, together with an indication of spike protein RBD and ACE2.

Another significant amino acid change was T478K, the one observed in all patients, leading to the substitution of hydrophobic threonine (T) to charged lysine (K), which was established to enhance spike protein surface electrostatic potential that might affect spike RBD affinity with human ACE-2 (30).

Studying the consequences of patients’ health conditions if infected with various mutated/unmutated SARS-CoV-2 would result in a deeper understanding of the outcomes of multiple mutations. However, we did not study any clinical consequences due to anonymized samples ruled out by the ethical committee. In such cases, using computer software is another reliable approach (31). We will use computational analysis, such as molecular docking or molecular dynamics simulations, to predict the consequences. Because of the large number of mutations in this work, we planned to conduct computational calculations as separate research for several mutations. For now, our study is limited to the outcomes and discussions obtained.

## Conclusion

This study’s comprehensive whole-genome sequence data facilitates tracing the origins and sources of circulating SARS-CoV-2 variants and the identification and comparative analysis of emerging variations within Uzbekistan and beyond. The genome sequencing of SARS-CoV-2, derived from specimens collected from infected individuals in Uzbekistan towards the conclusion of 2021, has been outlined in this study. This timeframe corresponds to the second wave of the coronavirus disease pandemic, which had attained nationwide dissemination. After acquiring these genomic sequences, efforts have been directed toward developing DNA-based and plant-based edible vaccines utilizing the identified SARS-CoV-2 strains prevalent in Uzbekistan. Presently, these vaccine candidates are undergoing clinical trial evaluations.

## Data availability statement

The datasets presented in this study can be found in online repositories. The names of the repository/repositories and accession number(s) can be found in the article/Supplementary material.

## Ethics statement

The studies involving humans were approved by the Dr. Kamal Rizaev, Chairman of the Ethics Committee of the Ministry of Health of the Republic of Uzbekistan; Mrs. Larisa Alieva, Private Clinic of BiogenMed. The studies were conducted in accordance with the local legislation and institutional requirements. The ethics committee/institutional review board waived the requirement of written informed consent for participation from the participants or the participants’ legal guardians/next of kin because In this study, verbal consent to participate in sample collection was preferred over written consent since patients were hesitant to sign any written document due to concerns about COVID-19 infection in their illness condition as well as a lack of knowledge of the genome sequencing investigation. Participants were explained that anonymized data, including age and biological sex, would be preserved for scientific reporting purposes. We informed all participants that the collected samples would be used for a “research purpose DNA sequencing project” exclusively, without disclosing their identities, following a scientific article publication without participants’ IDs. All patients in the sample collection provided verbal consent for voluntary participation. Moreover, in the internal government regulations, there was no demand to collect informed written consent from participants to publish results in a scientific article due to using samples taken for only a DNA sequencing experiment, which was non-invasive, and not related to any further downstream clinical procedures. We assure you that the samples were renumbered and de-identified so that no one, not even researchers knew the participant’s identity.

## Author contributions

MA: Conceptualization, Data curation, Funding acquisition, Methodology, Validation, Visualization, Writing – original draft, Writing – review & editing. MM: Methodology, Visualization. AY: Methodology, Visualization. AA: Methodology, Visualization, Writing – original draft, Writing – review & editing. BN: Methodology. DU: Methodology. SS: Methodology. KU: Methodology. AA: Methodology. ZB: Conceptualization, Data curation, Funding acquisition, Methodology, Visualization. IA: Supervision, Writing – original draft, Writing – review & editing.

## Footnotes

1
https://www.megasoftware.net

2
www.gisaid.org

3
https://swissmodel.expasy.org/interactive
