# Supplementary material for: Most accurate mutations in SARS-CoV-2 genomes identified in Uzbek patients show novel amino acid changes
Source: Front Med (Lausanne). 2024 May 31;11:1401655. doi: 10.3389/fmed.2024.1401655 (PMC11176497; doi:10.3389/fmed.2024.1401655)
Supplement: Supplementary file 1 [file Table_1.docx]

**Supplementary Table 1: One hundred thirty-four mutations were observed in seventeen SARS-CoV-2 viral genome sequences in samples from COVID-19 patients in Tashkent, Uzbekistan.**

| Mutation position | Genotype | GENE | HGVS_C | HGVS_P | MUTATION EFFECT | # of sequences carrying a mutation | Ion Torrent quality score (-10logP)*** |
| --- | --- | --- | --- | --- | --- | --- | --- |
| 210 | Homozygous | orf1ab | c.-56G>T | . | upstream_gene_variant | 16 | 2973.68 |
| 241 | Homozygous | orf1ab | c.-25C>T | . | upstream_gene_variant | 16 | 2895.83 |
| 1048 | Homozygous | orf1ab | c.783G>T | p.Lys261Asn | missense_variant | 5 | 2950.28 |
| 1186 | Homozygous | orf1ab | c.921G>T | p.Ala307Ala | synonymous_variant | 1 | 1717.33 |
| 1191 | Homozygous | orf1ab | c.926C>T | p.Pro309Leu | missense_variant | 3 | 2922.33 |
| 1243 | Homozygous | orf1ab | c.978T>C | p.Cys326Cys | synonymous_variant | 1 | 2900.46 |
| 1267 | Homozygous | orf1ab | c.1002C>T | p.Gly334Gly | synonymous_variant | 3 | 2938.64 |
| 1414 | Homozygous | orf1ab | c.1149T>C | p.Ser383Ser | synonymous_variant | 1 | 2819.33 |
| 1878 | Homozygous | orf1ab | c.1613C>T | p.Ser538Leu | missense_variant | 1 | 2877.91 |
| 2272 | Homozygous | orf1ab | c.2007G>T | p.Lys669Asn | missense_variant | 1 | 2785.46 |
| 3007 | Homozygous | orf1ab | c.2742T>C | p.Phe914Phe | synonymous_variant | 1 | 2780.79 |
| 3037 | Homozygous | orf1ab | c.2772C>T | p.Phe924Phe | synonymous_variant | 17 | 2979.59 |
| 3096 | Homozygous | orf1ab | c.2831C>T | p.Ser944Leu | missense_variant | 1 | 2819.4 |
| 3153 | Homozygous | orf1ab | c.2888G>T | p.Gly963Val | missense_variant | 2 | 2534.07 |
| 3695 | Homozygous | orf1ab | c.3430C>T | p.Leu1144Leu | synonymous_variant | 1 | 2931.02 |
| 4181 | Homozygous | orf1ab | c.3916G>T | p.Ala1306Ser | missense_variant | 12 | 2884.27 |
| 4560 | Homozygous | orf1ab | c.4295C>A | p.Ala1432Glu | missense_variant | 1 | 2976.31 |
| 5184 | Homozygous | orf1ab | c.4919C>T | p.Pro1640Leu | missense_variant | 5 | 2789.21 |
| 6376 | Homozygous | orf1ab | c.6111T>C | p.Asp2037Asp | synonymous_variant | 2 | 2926.62 |
| 6402 | Homozygous | orf1ab | c.6137C>T | p.Pro2046Leu | missense_variant | 12 | 2737.0 |
| 6539 | Homozygous | orf1ab | c.6274C>T | p.His2092Tyr | missense_variant | 2 | 2926.8 |
| 7124 | Homozygous | orf1ab | c.6859C>T | p.Pro2287Ser | missense_variant | 11 | 2909.72 |
| 7393 | Homozygous | orf1ab | c.7128G>T | p.Pro2376Pro | synonymous_variant | 1 | 2951.87 |
| 7869 | Homozygous | orf1ab | c.7604C>T | p.Ser2535Leu | missense_variant | 1 | 2780.91 |
| 8593 | Homozygous | orf1ab | c.8328T>C | p.Val2776Val | synonymous_variant | 1 | 2879.06 |
| 8986 | Homozygous | orf1ab | c.8721C>T | p.Asp2907Asp | synonymous_variant | 12 | 2894.6 |
| 9053 | Homozygous | orf1ab | c.8788G>T | p.Val2930Leu | missense_variant | 12 | 2937.9 |
| 9333 | Homozygous | orf1ab | c.9068C>T | p.Ala3023Val | missense_variant | 1 | 2942.92 |
| 9891 | Homozygous | orf1ab | c.9626C>T | p.Ala3209Val | missense_variant | 5 | 2968.13 |
| 10029 | Homozygous | orf1ab | c.9764C>T | p.Thr3255Ile | missense_variant | 13 | 2973.15 |
| 10323 | Homozygous | orf1ab | c.10058A>G | p.Lys3353Arg | missense_variant | 1 | 2067.45 |
| 10630 | Homozygous | orf1ab | c.10365A>G | p.Gln3455Gln | synonymous_variant | 1 | 2568.8 |
| 11020 | Homozygous | orf1ab | c.10755C>T | p.Leu3585Leu | synonymous_variant | 1 | 2668.95 |
| 11082 | Homozygous | orf1ab | c.10818delG | p.Leu3606fs | frameshift_variant | 1 | 1951.71 |
| 11195 | Homozygous | orf1ab | c.10930C>T | p.Leu3644Phe | missense_variant | 1 | 2855.48 |
| 11201 | Homozygous | orf1ab | c.10936A>G | p.Thr3646Ala | missense_variant | 12 | 2970.66 |
| 11332 | Homozygous | orf1ab | c.11067A>G | p.Val3689Val | synonymous_variant | 12 | 2970.54 |
| 11418 | Homozygous | orf1ab | c.11153T>C | p.Val3718Ala | missense_variant | 5 | 2792.54 |
| 11455 | Homozygous | orf1ab | c.11190C>T | p.Ala3730Ala | synonymous_variant | 1 | 2929.05 |
| 11514 | Homozygous | orf1ab | c.11249C>T | p.Thr3750Ile | missense_variant | 2 | 2878.04 |
| 11534 | Homozygous | orf1ab | c.11269G>A | p.Gly3757Ser | missense_variant | 1 | 2755.74 |
| 12946 | Homozygous | orf1ab | c.12681T>C | p.Tyr4227Tyr | synonymous_variant | 3 | 2718.97 |
| 13019 | Homozygous | orf1ab | c.12754C>T | p.Leu4252Leu | synonymous_variant | 2 | 2938.18 |
| 13168 | Homozygous | orf1ab | c.12903C>T | p.His4301His | synonymous_variant | 1 | 2826.82 |
| 13348 | Homozygous | orf1ab | c.13083G>T | p.Val4361Val | synonymous_variant | 1 | 2856.17 |
| 14103 | Homozygous | orf1ab | c.13839T>C | p.Asp4613Asp | synonymous_variant | 1 | 2804.55 |
| 14120 | Homozygous | orf1ab | c.13856C>T | p.Pro4619Leu | missense_variant | 1 | 2868.67 |
| 14262 | Homozygous | orf1ab | c.13998C>T | p.Asp4666Asp | synonymous_variant | 1 | 2900.36 |
| 14408 | Homozygous | orf1ab | c.14144C>T | p.Pro4715Leu | missense_variant | 16 | 2876.87 |
| 14408 | Homozygous | orf1ab | c.14144_14145delCTinsTC | p.Pro4715Leu | missense_variant | 1 | 2876.36 |
| 14622 | Homozygous | orf1ab | c.14358G>A | p.Thr4786Thr | synonymous_variant | 1 | 2864.3 |
| 14925 | Homozygous | orf1ab | c.14661C>T | p.Val4887Val | synonymous_variant | 6 | 2943.5 |
| 15451 | Homozygous | orf1ab | c.15187G>A | p.Gly5063Ser | missense_variant | 17 | 2977.34 |
| 15752 | Homozygous | orf1ab | c.15488C>T | p.Ala5163Val | missense_variant | 1 | 2897.99 |
| 16466 | Homozygous | orf1ab | c.16202C>T | p.Pro5401Leu | missense_variant | 17 | 2855.57 |
| 16912 | Homozygous | orf1ab | c.16648G>T | p.Val5550Leu | missense_variant | 1 | 2903.97 |
| 17523 | Homozygous | orf1ab | c.17259G>T | p.Met5753Ile | missense_variant | 1 | 2888.8 |
| 18176 | Homozygous | orf1ab | c.17912C>T | p.Pro5971Leu | missense_variant | 1 | 2525.58 |
| 18433 | Homozygous | orf1ab | c.18169G>A | p.Asp6057Asn | missense_variant | 1 | 2802.93 |
| 19220 | Homozygous | orf1ab | c.18956C>T | p.Ala6319Val | missense_variant | 12 | 2976.06 |
| 19735 | Homozygous | orf1ab | c.19471G>T | p.Asp6491Tyr | missense_variant | 1 | 2856.21 |
| 20068 | Homozygous | orf1ab | c.19804G>T | p.Gly6602Cys | missense_variant | 1 | 2865.33 |
| 20262 | Homozygous | orf1ab | c.19998A>G | p.Leu6666Leu | synonymous_variant | 3 | 2965.14 |
| 20320 | Homozygous | orf1ab | c.20056C>T | p.His6686Tyr | missense_variant | 2 | 2872.72 |
| 20573 | Homozygous | orf1ab | c.20309T>C | p.Val6770Ala | missense_variant | 2 | 2935.35 |
| 20578 | Homozygous | orf1ab | c.20314G>T | p.Val6772Leu | missense_variant | 2 | 2972.18 |
| 20670 | Homozygous | orf1ab | c.20406G>A | p.Ala6802Ala | synonymous_variant | 2 | 2865.07 |
| 20841 | Homozygous | orf1ab | c.20577A>G | p.Val6859Val | synonymous_variant | 1 | 2901.85 |
| 20937 | Homozygous | orf1ab | c.20673G>T | p.Thr6891Thr | synonymous_variant | 1 | 2936.66 |
| 21088 | Homozygous | orf1ab | c.20824G>T | p.Asp6942Tyr | missense_variant | 1 | 2751.85 |
| 21137 | Homozygous | orf1ab | c.20873A>G | p.Lys6958Arg | missense_variant | 6 | 2622.66 |
| 21618 | Homozygous | S | c.56C>G | p.Thr19Arg | missense_variant | 17 | 2959.25 |
| 21987 | Homozygous | S | c.425G>A | p.Gly142Asp | missense_variant | 10 | 2934.02 |
| 22028 | Homozygous | S | c.467_472delAGTTCA | p.Glu156_Arg158delinsGly | disruptive_inframe_deletion | 17 | 2958.81 |
| 22208 | Homozygous | S | c.646C>T | p.Leu216Phe | missense_variant | 1 | 2253.51 |
| 22227 | Homozygous | S | c.665C>T | p.Ala222Val | missense_variant | 2 | 2919.3 |
| 22498 | Homozygous | S | c.936C>T | p.Ile312Ile | synonymous_variant | 1 | 2971.42 |
| 22917 | Homozygous | S | c.1355T>G | p.Leu452Arg | missense_variant | 17 | 2977.57 |
| 22995 | Homozygous | S | c.1433C>A | p.Thr478Lys | missense_variant | 17 | 2979.66 |
| 23403 | Homozygous | S | c.1841A>G | p.Asp614Gly | missense_variant | 17 | 2970.43 |
| 23557 | Homozygous | S | c.1995C>T | p.Pro665Pro | synonymous_variant | 1 | 2584.54 |
| 23604 | Homozygous | S | c.2042C>G | p.Pro681Arg | missense_variant | 17 | 2956.33 |
| 23821 | Homozygous | S | c.2259G>A | p.Leu753Leu | synonymous_variant | 1 | 2907.69 |
| 23950 | Homozygous | S | c.2388T>C | p.Asp796Asp | synonymous_variant | 1 | 2402.2 |
| 24095 | Homozygous | S | c.2533G>T | p.Ala845Ser | missense_variant | 1 | 2918.66 |
| 24110 | Homozygous | S | c.2548A>C | p.Ile850Leu | missense_variant | 6 | 2980.96 |
| 24410 | Homozygous | S | c.2848G>A | p.Asp950Asn | missense_variant | 17 | 2970.83 |
| 24745 | Homozygous | S | c.3183C>T | p.Val1061Val | synonymous_variant | 2 | 2937.14 |
| 25323 | Homozygous | S | c.3761G>T | p.Cys1254Phe | missense_variant | 1 | 2824.08 |
| 25352 | Homozygous | S | c.3790G>T | p.Val1264Leu | missense_variant | 1 | 2925.2 |
| 25439 | Homozygous | ORF3a | c.47A>C | p.Lys16Thr | missense_variant | 6 | 2966.3 |
| 25469 | Homozygous | ORF3a | c.77C>T | p.Ser26Leu | missense_variant | 17 | 2911.88 |
| 25511 | Homozygous | ORF3a | c.119C>T | p.Ser40Leu | missense_variant | 1 | 2945.05 |
| 25571 | Homozygous | ORF3a | c.179C>T | p.Ser60Phe | missense_variant | 2 | 2530.94 |
| 25638 | Homozygous | ORF3a | c.246C>T | p.Asn82Asn | synonymous_variant | 1 | 2851.94 |
| 25690 | Homozygous | ORF3a | c.298G>T | p.Gly100Cys | missense_variant | 1 | 2900.3 |
| 25775 | Homozygous | ORF3a | c.383G>T | p.Trp128Leu | missense_variant | 1 | 2651.57 |
| 25996 | Homozygous | ORF3a | c.604G>T | p.Val202Leu | missense_variant | 1 | 2747.34 |
| 26101 | Homozygous | ORF3a | c.709G>T | p.Val237Phe | missense_variant | 1 | 2819.39 |
| 26171 | Homozygous | ORF3a | c.779T>A | p.Met260Lys | missense_variant | 1 | 2897.25 |
| 26195 | Homozygous | ORF3a | c.803C>T | p.Thr268Met | missense_variant | 1 | 2264.05 |
| 26622 | Homozygous | M | c.100C>T | p.Leu34Phe | missense_variant | 1 | 1522.2 |
| 26767 | Homozygous | M | c.245T>C | p.Ile82Thr | missense_variant | 17 | 2981.88 |
| 26985 | Homozygous | M | c.463C>T | p.His155Tyr | missense_variant | 1 | 2843.91 |
| 27223 | Homozygous | ORF6 | c.22C>G | p.Gln8Glu | missense_variant | 1 | 1028.06 |
| 27526 | Homozygous | ORF7a | c.133_134delCCinsTT | p.Pro45Leu | missense_variant | 1 | 2938.45 |
| 27527 | Homozygous | ORF7a | c.134C>T | p.Pro45Leu | missense_variant | 4 | 2963.09 |
| 27573 | Homozygous | ORF7a | c.180C>T | p.Ser60Ser | synonymous_variant | 1 | 2878.82 |
| 27583 | Homozygous | ORF7a | c.190G>T | p.Ala64Ser | missense_variant | 2 | 2860.94 |
| 27638 | Homozygous | ORF7a | c.245T>C | p.Val82Ala | missense_variant | 16 | 2925.75 |
| 27739 | Homozygous | ORF7a | c.346C>T | p.Leu116Phe | missense_variant | 3 | 2975.07 |
| 27752 | Homozygous | ORF7a | c.359C>T | p.Thr120Ile | missense_variant | 16 | 2965.35 |
| 27874 | Homozygous | ORF8 | c.-20C>T | . | upstream_gene_variant | 11 | 2926.06 |
| 28096 | Homozygous | ORF8 | c.203A>T | p.Lys68Ile | missense_variant | 1 | 2860.46 |
| 28247 | Homozygous | ORF8 | c.355_360delGATTTC | p.Asp119_Phe120del | conservative_inframe_deletion | 17 | 2724.19 |
| 28270 | Homozygous | N | c.-3delA | . | upstream_gene_variant | 17 | 1923.53 |
| 28378 | Homozygous | N | c.105G>A | p.Ala35Ala | synonymous_variant | 1 | 2888.82 |
| 28396 | Homozygous | N | c.123G>A | p.Arg41Arg | synonymous_variant | 1 | 2799.86 |
| 28461 | Homozygous | N | c.188A>G | p.Asp63Gly | missense_variant | 17 | 2975.39 |
| 28473 | Homozygous | N | c.200C>T | p.Pro67Leu | missense_variant | 1 | 2883.4 |
| 28791 | Homozygous | N | c.518C>T | p.Ala173Val | missense_variant | 1 | 2738.92 |
| 28881 | Homozygous | N | c.608G>T | p.Arg203Met | missense_variant | 17 | 2840.33 |
| 28916 | Homozygous | N | c.643G>T | p.Gly215Cys | missense_variant | 12 | 2972.15 |
| 29260 | Homozygous | N | c.987G>A | p.Thr329Thr | synonymous_variant | 1 | 2938.82 |
| 29353 | Homozygous | N | c.1080C>T | p.Tyr360Tyr | synonymous_variant | 1 | 2955.45 |
| 29358 | Homozygous | N | c.1085C>T | p.Thr362Ile | missense_variant | 1 | 2860.8 |
| 29402 | Homozygous | N | c.1129G>T | p.Asp377Tyr | missense_variant | 17 | 2960.15 |
| 29425 | Homozygous | N | c.1152G>C | p.Gln384His | missense_variant | 2 | 2941.5 |
| 29427 | Homozygous | N | c.1154G>A | p.Arg385Lys | missense_variant | 2 | 2930.92 |
| 29614 | Homozygous | ORF10 | c.57C>T | p.Cys19Cys | synonymous_variant | 1 | 2890.25 |
| 29684 | Homozygous | S | c.*4300A>G | . | downstream_gene_variant | 1 | 2680.89 |
| 29692 | Homozygous | S | c.*4308G>T | . | downstream_gene_variant | 2 | 2870.99 |
| 29736 | Homozygous | S | c.*4352G>T | . | downstream_gene_variant | 1 | 2856.21 |
| 29742 | Homozygous | S | c.*4358G>T | . | downstream_gene_variant | 17 | 2969.62 |
