# Supplementary material for: Most accurate mutations in SARS-CoV-2 genomes identified in Uzbek patients show novel amino acid changes
Source: Front Med (Lausanne). 2024 May 31;11:1401655. doi: 10.3389/fmed.2024.1401655 (PMC11176497; doi:10.3389/fmed.2024.1401655)
Supplement: Supplementary file 2 [file Table_2.docx]

**S2 Table. Nucleotide mutations of the matrix (M), nucleocapsid (N), and spike (S) regions of Uzbekistan SARS-CoV-2 sequences based on comparison to the reference sequence** (GenBank reference sequence accession number NC_045512.2).

| # | **Nucleotide position** | **Gene** | **Reference nucleotide** | **Sequenced nucleotide** | **Mutation type** | **Nucleotide change** | **Amino acid change (position)** |
| --- | --- | --- | --- | --- | --- | --- | --- |
| 1 | *26622* | M | C | T | missense_variant | C→T | L34F |
| 2 | *26767* | M | T | C | missense_variant | T→C | I82T |
| 3 | *26985* | M | C | T | missense_variant | C→T | H155Y |
| 4 | *28461* | N | A | G | missense_variant | A→G | D63G |
| 5 | *28473* | N | C | T | missense_variant | C→T | P67L |
| 6 | *28791* | N | C | T | missense_variant | C→T | A173V |
| 7 | *28881* | N | G | T | missense_variant | G→T | R203M |
| 8 | *28916* | N | G | T | missense_variant | G→T | G215C |
| 9 | *29358* | N | C | T | missense_variant | C→T | T362I |
| 10 | *29402* | N | G | T | missense_variant | G→T | D377Y |
| 11 | *29425* | N | G | C | missense_variant | G→C | Q384H |
| 12 | *29427* | N | G | A | missense_variant | G→A | R385K |
| 13 | *28378* | N | G | A | synonymous_variant | G→A | A35A |
| 14 | *28396* | N | G | A | synonymous_variant | G→A | R41R |
| 15 | *29260* | N | G | A | synonymous_variant | G→A | T329T |
| 16 | *29353* | N | C | T | synonymous_variant | C→T | Y360Y |
| 17 | *28270* | N | A | . | upstream_gene_variant | *3del→A | . |
| 18 | *21618* | S | C | G | missense_variant | C→G | T19R |
| 19 | *21987* | S | G | A | missense_variant | G→A | G142D |
| 20 | *22208* | S | C | T | missense_variant | C→T | L216F |
| 21 | *22227* | S | C | T | missense_variant | C→T | A222V |
| 22 | *22917* | S | T | G | missense_variant | T→G | L452R |
| 23 | *22995* | S | C | A | missense_variant | C→A | T478K |
| 24 | *23403* | S | A | G | missense_variant | A→G | D614G |
| 25 | *23604* | S | C | G | missense_variant | C→G | P681R |
| 26 | *24095* | S | G | T | missense_variant | G→T | A845S |
| 27 | *24110* | S | A | C | missense_variant | A→C | I850L |
| 28 | *24410* | S | G | A | missense_variant | G→A | D950N |
| 29 | *25323* | S | G | T | missense_variant | G→T | C1254F |
| 30 | *25352* | S | G | T | missense_variant | G→T | V1264L |
| 31 | *22498* | S | C | T | synonymous_variant | C→T | I312I |
| 32 | *23557* | S | C | T | synonymous_variant | C→T | P665P |
| 33 | *23821* | S | G | A | synonymous_variant | G→A | L753L |
| 34 | *23950* | S | T | C | synonymous_variant | T→C | D796D |
| 35 | *24745* | S | C | T | synonymous_variant | C→T | V1061V |
| 36 | *22028* | S | AGTTCA | . | disruptive_inframe_deletion | *467_472 | . |
| 37 | *29684* | S | A | G | downstream_gene_variant | A→G | . |
| 38 | *29692* | S | G | T | downstream_gene_variant | G→T | . |
| 39 | *29736* | S | G | T | downstream_gene_variant | G→T | . |
| 40 | *29742* | S | G | T | downstream_gene_variant | G→T | . |
